# Electric Control of Photonic Spin Hall Effect in Surface Plasmon Resonance Systems for Multi-Functional Sensing

**DOI:** 10.3390/s25175383

**Published:** 2025-09-01

**Authors:** Jiaye Ding, Ruizhao Li, Jie Cheng

**Affiliations:** School of Science, Jiangsu Province Engineering Research Center of Low Dimensional Physics and New Energy, Nanjing University of Posts and Telecommunications, Nanjing 210023, China; a1452516198@163.com (J.D.); 1022082203@njupt.edu.cn (R.L.)

**Keywords:** photonic spin Hall effect, surface plasmon resonance, electric control, sensor

## Abstract

The photonic spin Hall effect (PSHE) has emerged as a powerful metrological approach for precision measurements. Dynamic manipulation of PSHE through external stimuli could substantially expand its applications. In this work, we present a simple and active modulation scheme for PSHE in a surface plasmon resonance (SPR) structure by exploiting electric-field-tunable refractive indices of electro-optic materials. By applying an electric field, the enhancement of PSHE spin shifts is observed, and the dual-field control can further amplify these spin shifts through synergistic effects in this SPR structure. Notably, various operation modes of external electric field enable the real-time switching between two high-performance sensing functionalities (refractive index detection and angle measurement). Therefore, our designed PSHE sensor based on SPR structure with a simple structure of only three layers not only makes up for the complex structure in multi-functional sensors, but more importantly, this platform establishes a new paradigm for dynamic PSHE manipulation while paving the way for advanced multi-functional optical sensing technology.

## 1. Introduction

The photonic spin Hall effect (PSHE) is a fascinating optical phenomenon, in which left- and right-handed circularly polarized components of a linearly polarized light split undergo transverse separation (perpendicular to the plane of incidence) when the beam is reflected or transmitted at an interface [1,2,3]. The PSHE can be considered as an optical analogy of the spin Hall effect (SHE) in electronic systems, where spin-polarized photons and a refractive index gradient are analogous to spin-polarized electrons and an electric potential gradient, respectively [4,5]. This effect arises from the interaction between the photon-spin (polarization) and the trajectory (extrinsic orbital angular momentum) [5]. Because of its unique physical mechanism, the PSHE has attracted much attention and exhibited potential applications in nanophotonics, particularly in precision measurement and refractive index sensing [6,7,8,9,10,11,12,13,14,15].

However, the PSHE is a weak effect, whose spin-dependent spin shift is usually limited to the sub-wavelength scale [16]. Therefore, it cannot be directly observed with conventional experimental methods, and its successful detection should be incorporated with the weak measurement technique [16]. To overcome this limitation and facilitate its application, various strategies have been put forward to enhance the PSHE and achieve larger spin shifts. Firstly, surface plasmon resonance (SPR) [17]: SPR-induced absorption creates a reflectance minimum at the SPR angle. The spin shifts in PSHE can be dramatically amplified, due to the minimal $\left|r_{p}\right|$ and maximal value of $\frac{\left|r_{s}\right|}{\left|r_{p}\right|}$. Secondly, Brewster angle [18]: When altering the incident angle, the spin-dependent shift undergoes sign reversal on either side of the Brewster angle. Consequently, slight angular variations enable efficient modulation of the reflected PSHE. The generation of giant spin-splitting values near the Brewster angle originates from the strong spin–orbit interaction. Thirdly, resonant optical tunneling effect [19]: Enhancing the refractive index gradient amplifies the potential barrier profile throughout the structure, thereby increasing the magnitude of PSHE shifts. Recently, researchers greatly enhance the PSHE empowered by polarization-sensitive quasi-bound states in the continuum [20,21,22] and polarization-sensitive photonic bandgaps [23]. Among them, the SPR method has been widely adopted to enhance the PSHE and design the optical devices, owing to its structural simplicity. There is a momentum mismatch between the incident photons and the surface plasmon polariton (SPP) mode, which can be overcome by coupling the incident field to surface modes through a diffraction grating, or high index prism. By doing so, the matching between the wave vector of incident light and SPP can be realized, and then the direct optical excitation of SPP modes can be achieved. For instance, in a prism-coupled SPR structure (glass–gold–air), a spin shift of about 3 μm is obtained [17]. Further structural innovations have led to even greater enhancements in spin shifts. A bimetallic structure of prism–silver–gold–silica achieves a spin shift of 5.34 μm [24]. By taking advantage of long-range SPR (the coupling of two surface plasmon polaritons) in a prism–silica–gold–silica structure, the spin shift reaches up to 7.85 μm [25]. Remarkably, a guided-wave SPR design incorporating a high-refractive index dielectric (silicon) on silver film (i.e., prism–silver–silicon–air structure) yields a giant spin shift of 11 μm [26]. While these methods provide an opportunity for enhancing the PSHE and show great potential for new types of nanophotonic devices, they rely on the static structural modifications. Therefore, integrating dynamically tunable PSHE into spin-based nanophotonic systems remains a critical goal for future advancements.

For dynamic and flexible manipulation of the PSHE, post-fabrication methods utilizing external stimuli have demonstrated the significant potential [12,27,28,29,30,31,32,33]. In view of the intimate relationship between PSHE and refractive index gradient, extremely small variations in refractive index can induce significant changes in spin-dependent shifts. This principle has been successfully implemented through various external modulation techniques. Thermal [27,28], electrical [29], magnetic [30], and optical pumping [31], have been employed to tune the refractive indices of bulk or interface materials, thereby enabling active control of PSHE and device performance. For example, Dong et al. demonstrated optical pumping as an effective approach for dynamically manipulating PSHE in the graphene–dielectric structure [31]. Aided by the bias-assisted light, the PSHE of glucose sensor can be actively manipulated, enabling the modulation of sensing performance synchronously [32,33]. Very recently, Cheng et al. and Cao et al. have exploited the thermally induced insulator–metal transition of VO_2_ to realize dynamic PSHE modulation, with potential applications in the two-digit binary code conversion and multi-functional sensing [27,28].

For the electro-optic (EO) materials, modifying their refractive indices can also be accomplished by means of an external electric field, a widely explored technique [34,35,36]. Based on the linear change in the refractive indices caused by the application of an electric field, the Pockels EO effect offers a new paradigm for dynamic modulation of PSHE. In this work, we propose and theoretically investigate the integration of external electric fields into the SPR structure to achieve dynamic control of PSHE. Furthermore, the exploration of its multi-functional sensing applications based on the SPR structure containing EO materials is also conducted.

## 2. Theory and Model

**Figure 1:**
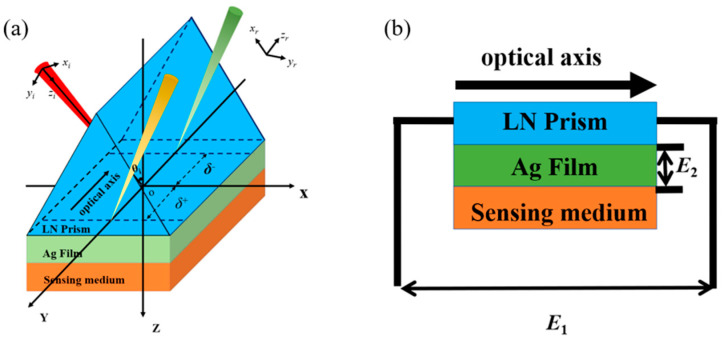
Schematic diagram of Kretschmann configuration with the applied electric field. (**a**) three-dimensional image, (**b**) two-dimensional image and the direction of the applied electric field.

The proposed Kretschmann configuration, illustrated in Figure 1, consists of lithium niobate (LN) prism, silver (Ag) thin film, and sensing medium. Both the LN prism and sensing medium are modeled as semi-infinite domains. A He-Ne laser light source provides the incident illumination at a wavelength of 632.8 nm. The Ag film is characterized by a complex permittivity $\varepsilon_{20}=\;-16.9+1.21i$ [35] and a thickness *d* of 48 nm. As a typical EO material, LN exhibits anisotropic properties. As shown in Figure 1, the optical axis of LN is perpendicular to the incident plane, and the external electric field $E_{1}$ is applied parallel to the optical axis. In this work, the incident light for generating the PSHE is TM-polarized wave, then the electric field remains orthogonal to LN’s optical axis, enabling treatment of the system as isotropic under these conditions. Based on the above analysis, the LN in this work can be regarded as an isotropic material. A secondary electric field $E_{2}$ is applied along the z-direction to the Ag layer, which induces surface charge polarization and results in modifications of electrical and optical properties.

Due to the EO effect, the changes in refractive indices of the LN prism and Ag film are given by [34,35]

(1) $${∆n}_{1}=-\frac{1}{2}n_{1}^{3}{\gamma_{13}E}_{1}$$

(2) $${∆n}_{2}=-\frac{1}{2}n_{2}^{3}\gamma_{33}E_{2}$$

where $n_{1}(n_{1}\;=\;2.28)$ and $n_{2}$ represent the refractive indices of LN prism and Ag film without an applied electric field, respectively. $\gamma_{13}(\gamma_{13}=9.25\;pm/V)$ [37] and $\gamma_{33}(\gamma_{33}=3.76+0.27i\;pm/V)$ [35] are the component of EO coefficients. $E_{1}$ and $E_{2}$ are the applied electric field on the LN prism and Ag film, respectively. For a given value of the electric field, the effective permittivity of LN prism and Ag film can be written as $\varepsilon_{1}\;=\;{\left(n_{1}+{\Delta n}_{1}\right)}^{2}$ and $\varepsilon_{2}{=(n_{2}+{\Delta n}_{2})}^{2}$.

A monochromatic Gaussian beam is incident from the LN prism, and it will be reflected by the Ag layer due to the excitation of SPR. Simultaneously, the PSHE occurs resulting in the splitting of left- and right-handed circularly polarized components. In order to calculate the spin shifts of PSHE, a general beam propagation model using angular spectrum theory is employed. The incident Gaussian beam can be expressed as [38]

(3) $${\tilde{E}}_{i}=\frac{w_{0}}{\sqrt{2\pi }}exp\left[-\frac{w_{0}^{2}(k_{ix}^{2}+k_{iy}^{2})}{4}\right]$$

in which $w_{0}$ is the beam waist. In the spin basis set, the angular spectrum of incident beam can be written as ${\tilde{E}}_{i}^{H}=({\tilde{E}}_{i+}+{\tilde{E}}_{i-})/\sqrt{2}$ and ${\tilde{E}}_{i}^{V}=i({\tilde{E}}_{i-}-{\tilde{E}}_{i+})/\sqrt{2}$. Here, H and V denote the horizontal and vertical polarization states, respectively. ${\tilde{E}}_{i+}$ and ${\tilde{E}}_{i-}$ present the left- and right-handed circularly polarized components. The relation between the angular spectra of reflected and incident beam can be described as [38]

(4) $$\left[\begin{array}{c} {\tilde{E}}_{r}^{H}\\ {\tilde{E}}_{r}^{V} \end{array}\right]=\left[\begin{array}{cc} r_{p} & \frac{k_{ry}cot\theta_{i}(r_{p}+r_{s})}{k_{0}}\\ -\frac{k_{ry}cot\theta_{i}(r_{p}+r_{s})}{k_{0}} & r_{s} \end{array}\right]\left[\begin{array}{c} {\tilde{E}}_{i}^{H}\\ {\tilde{E}}_{i}^{V} \end{array}\right]$$

Here, $r_{s}$ and $r_{p}$ are the Fresnel reflection coefficients of *s* and *p* waves, which can be calculated by the transfer matrix method [38]

(5) $$M=T_{01}P_{1}T_{12}P_{2}\cdots P_{m-2}T_{m-2,m-1}P_{m-1}T_{m-1,m}$$

where

(6) $$T_{m-1,m}=\frac{1}{t_{m-1,m}}\left[\begin{array}{cc} 1 & r_{m-1,m}\\ r_{m-1,m} & 1 \end{array}\right]$$

is the transformation matrix from layer (*m* − 1) to layer *m*, and

(7) $$P_{m}=\left[\begin{array}{cc} exp(-ik_{mz}d_{m}) & 0\\ 0 & exp(ik_{mz}d_{m}) \end{array}\right]$$

is the transmission matrix for layer *m*. Here, $d_{m}$ is the thickness of layer *m*. $r_{m-1,m}$ and $t_{m-1,m}$ are reflection and transmission coefficients from layer (*m* − 1) to layer *m*, respectively. The Fresnel reflection coefficients can be given by the elements of transfer matrix:

(8) $$r_{p,s}\;=\;\frac{M_{21}}{M_{11}}$$

For a Gaussian incident light, the two circular components of the reflected field can be given as [25]

(9) $$E_{\pm }^{H,V}\propto \frac{w_{0}}{w}exp[-\frac{x_{r}^{2}+y_{r}^{2}}{w}][r_{p}-\frac{2ix_{r}}{k_{0}w}\cdot \frac{\partial r_{p}}{\partial \theta }\mp \frac{2y_{r}\cot\theta_{i}}{k_{0}w}\left(r_{p}+r_{s}\right)]$$

in which $w=w_{0}{\left[1+{\left(2z_{r}/k_{0}{w_{0}}^{2}\right)}^{2}\right]}^{1/2}$, and ± denotes the left and right circularly polarized components. Then, the spin shift of the reflected light can be defined as

(10) $$\delta_{\pm }^{H,V}=\frac{\iint y{\left|E_{\pm }^{H,V}\right|}^{2}dxdy}{\iint {\left|E_{\pm }^{H,V}\right|}^{2}dxdy}$$

If we expand the Fresnel coefficients to first order in a Taylor series, ignoring higher-order infinitesimal quantities, the spin-dependent transverse shifts can be derived as [25]

(11) $$\delta_{\pm }^{H}=\mp \frac{k_{0}{w_{0}}^{2}Re(1+r_{s}/r_{p})\cot\theta_{i}}{{k_{0}}^{2}{w_{0}}^{2}+{\left|\frac{\partial lnr_{p}}{\partial \theta_{i}}\right|}^{2}+{\left|(1+r_{s}/r_{p})\cot\theta_{i}\right|}^{2}}$$

(12) $$\delta_{\pm }^{V}=\mp \frac{k_{0}{w_{0}}^{2}Re(1+r_{p}/r_{s})\cot\theta_{i}}{{k_{0}}^{2}{w_{0}}^{2}+{\left|\frac{\partial lnr_{s}}{\partial \theta_{i}}\right|}^{2}+{\left|(1+r_{p}/r_{s})\cot\theta_{i}\right|}^{2}}$$

If the reflection coefficients $r_{s}$ and $r_{p}$ are weakly dependent on the incident angle $\theta_{i}$, the FWHM of the reflection coefficient is broad. In this case, the transverse displacement of the reflected beam can be approximated using only the zero-order Taylor expansion in Equations (11) and (12), then the spin shift of the reflected light can be defined as [38]

(13) $$\delta_{\pm }^{H}=\mp \frac{\lambda }{2\pi }[1+\frac{\left|r_{s}\right|}{\left|r_{p}\right|}cos(\varphi_{s}-\varphi_{p})]cot\theta_{i}$$

(14) $$\delta_{\pm }^{V}=\mp \frac{\lambda }{2\pi }[1+\frac{\left|r_{p}\right|}{\left|r_{s}\right|}cos(\varphi_{p}-\varphi_{s})]cot\theta_{i}$$

where $r_{p,s}=|r_{p,s}|exp(i\varphi_{p,s})$, and  λ  is wavelength of the incident beam. Therefore, Equations (13) and (14) are only applicable when the beam waist is sufficiently wide.

The refractive indices of both LN prism and Ag film exhibit electric-field-dependent tunability through the EO effect, which will affect the Fresnel coefficients and spin shifts of PSHE. This work systematically investigates the modulation of PSHE in the proposed

SPR structure under different values of external electric field. Furthermore, the multi-functional sensing applications (including both refractive index and angle detections) based on the electrical-controlled PSHE are also explored.

## 3. Simulation Results and Analysis

**Figure 2:**
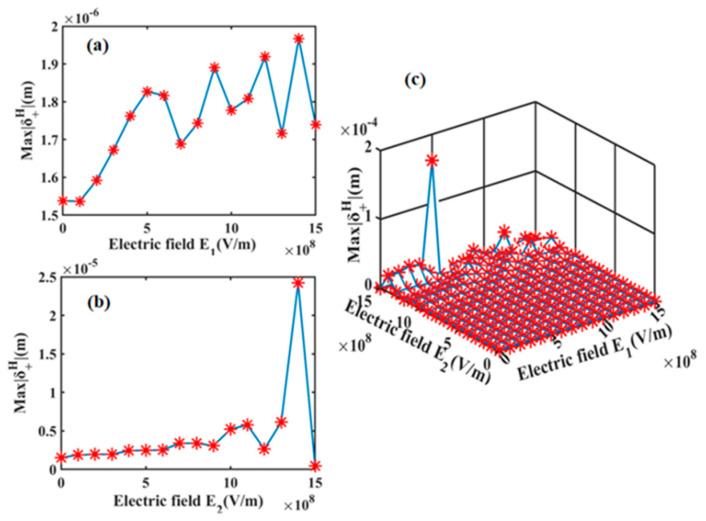
The maximal spin shift ${Max|\delta }_{\;+}^{H}|$ of PSHE under different conditions of applied electric field: (**a**) $E_{1}$-only, (**b**) $E_{2}$-only, and (**c**) dual-field ($E_{1}$ + $E_{2}$).

As clearly seen in Equations (1) and (2), the refractive indices of both the LN prism and Ag film exhibit a linear dependence on the applied electric field; therefore, the spin shifts of PSHE would be flexibly modulated via the electric field. Here, the two external electric fields are independent, thus $E_{1}$ and $E_{2}$ can be applied individually or concurrently, yielding four distinct operational modes: zero-field, $E_{1}$-only, $E_{2}$-only, and dual-field ($E_{1}$ + $E_{2}$). The applied electric fields vary from 0 to 15 × 10^8^ V/m in increments of 1 × 10^8^ V/m. This order of magnitude for electric field has been widely used in other reports [34]. Each 1 × 10^8^ V/m increment of electric field induces the appreciable refractive index changes of 0.005 (LN prism) and −0.005–0.0131*i* (Ag film), respectively. Figure 2 shows the maximal spin shift ${Max|\delta }_{\;+}^{H}|$ under different electric field conditions. In this calculation, the refractive index of sensing medium is set to be 1. When only the electric field $E_{1}$ is applied on LN prism (seen in Figure 2a), there is a fluctuant ascending tendency of ${Max|\delta }_{\;+}^{H}|$ to the $E_{1}$. In other words, the application of electric field on LN prism can enhance the spin shift of PSHE to a certain extent, and the maximal value of spin shift occurs at the electric field strength $E_{1}$ of 14 × 10^8^ V/m. The similar enhancement trend of ${Max|\delta }_{\;+}^{H}|$ is also observed for $E_{2}$ application (in Figure 2b). More significantly, when we adopt the control strategy of dual electric fields (in Figure 2c, $E_{1}$ = 5 × 10^8^ V/m and $E_{2}$ = 15 × 10^8^ V/m), the spin shift of PSHE has a further increase, which fully demonstrates the cooperative enhancement of PSHE exceeding individual field effects.

**Figure 3:**
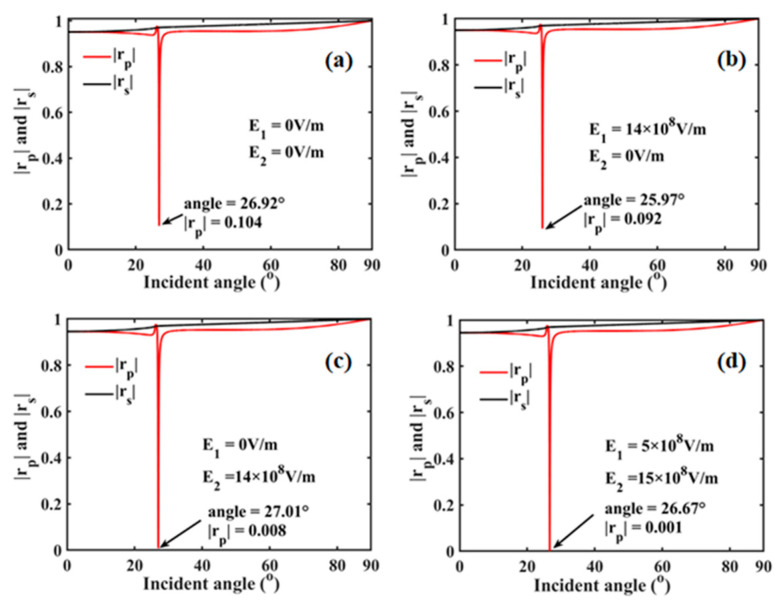
The Fresnel coefficient coefficients (${|r}_{s}|,{|r}_{p}|$) versus the incident angle under different conditions of applied electric field. (**a**) zero-field, (**b**) $E_{1}$-only, (**c**) $E_{2}$-only, and (**d**) dual-field ($E_{1}$ + $E_{2}$).

According to Equations (9) and (10), the spin shift of PSHE is strongly dependent on the Fresnel reflection coefficients. Specifically, the value of $\delta^{H}$ can be enhanced by enlarging the differences between $r_{s}$ and $r_{p}$, while keeping $r_{p}$ as small as possible. To elucidate how the PSHE can be tuned by the electric field, the Fresnel coefficients (${|r}_{s}|,{|r}_{p}|$) versus the incident angle under different conditions of applied electric fields are plotted in Figure 3. Without an electric field, ${|r}_{s}|$ remains nearly constant at a large value (~1) across all incident angles. In contrast, there is a narrow dip (~0.104) at 26.92° in the ${|r}_{p}|$ reflection curve, corresponding to the notable SPR effect in this Kretschmann configuration [17]. When an electric field is applied solely to LN prism or Ag film, the minimum value of ${|r}_{p}|$ decreases to 0.092 (at 25.97°) and 0.008 (at 27.01°), respectively. By introducing the synergistic regulation of dual electric field, ${|r}_{p}|$ reaches an even lower minimum of 0.001 at 26.67°, indicating the significant modulation effect of external electric fields on the SPR system. To sum up, the application of electric field is beneficial to decrease the dip of ${|r}_{p}|$ reflection curve. Especially in the conditions of $E_{2}$-only and dual-field control, the dip of ${|r}_{p}|$ reduces by one or two orders of magnitude, respectively. This substantial decrease in ${|r}_{p}|$ contributes to the enhancement of spin shifts in PSHE.

**Figure 4:**
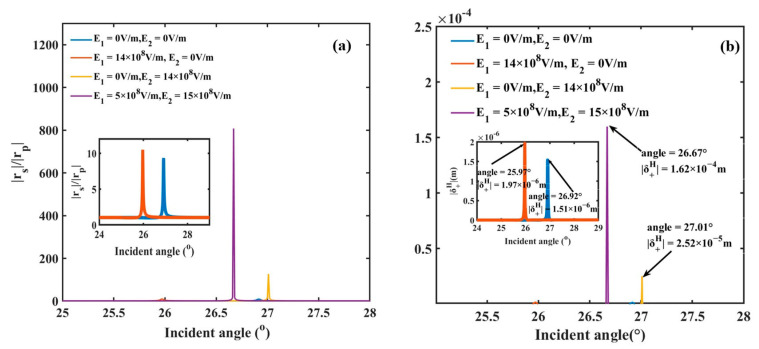
(**a**) The Fresnel reflection coefficients ratio ${|r}_{s}|/{|r}_{p}|$ and (**b**) spin shift ${|\delta }_{\;+}^{H}|$ under different conditions of applied electric field.

In the Kretschmann configuration, the excitation of SPR is characterized by a sharp dip in the ${|r}_{p}|$ reflection curve near the resonance angle [17]. Introducing external electric fields deepens this dip and significantly increases the Fresnel reflection coefficients ratio ${|r}_{s}|/{|r}_{p}|$. Without an electric field (in Figure 4a), the ratio ${|r}_{s}|/{|r}_{p}|$ at the resonance angle of 26.92° is less than 10. When only $E_{1}$ or $E_{2}$ is applied, the ratio undergoes a slight increase. Under the condition of dual electric field control, the ratio dramatically enhances to 800 at the resonance angle, which is 80 times larger than that without an electric field. Accordingly, the spin shift ${|\delta }_{\;+}^{H}|$ of PSHE under different conditions of applied electric field is also exhibited in Figure 4b. It is clearly seen that the maximal value of spin shift is just 1.51 μm when no electric field is applied. By introducing the external electric field, there is an enhancement of spin shift; for the application of $E_{1}$-only or $E_{2}$-only, the maximal values of spin shift are up to 1.97 μm and 25.2 μm, respectively. Most strikingly, dual-field control yields a maximal spin shift of 162 μm. Compared to the condition without an electric field, this value is enhanced by two orders of magnitude, providing an effective strategy for enhancing PSHE spin shifts in SPR structures containing EO materials.

With the rapid advancement of high-precision scientific research, the requirements for highly sophisticated and multi-functional sensors become increasingly stringent. Recently, PSHE-based sensors have received a great deal of attention due to their promising advantages, such as high sensitivity, real-time detection capability, and rapid response [12,13,14,15]. Considering the dynamic tunable and enhanced PSHE via the external electric field, the multi-functional sensing application (including both refractive index and angle detections) based on this SPR structure containing EO materials is theoretically proposed. Here, under the conditions of zero-field and $E_{1}$-only application, the spin shifts of PSHE in this SPR structure exhibit remarkable sensitivity to the refractive index of sensing medium, making it suitable for gas pollutant detection. Meanwhile, when $E_{2}$-only application or dual electric field control, the system can be considered as an angle sensor. Therefore, based on the various operational modes of the external electric field, this PSHE sensor is expected to realize the multi-functional sensing. Generally, the sensitivity is a fundamental and important parameter that characterizes the sensing performance. For refractive index sensing, the intensity sensitivity can be defined as $S_{\delta }=\frac{\Delta \delta }{\Delta n}$, where Δδ is the change in spin shifts (under a fixed incident angle) caused by a refractive index variation Δn of sensing medium [24]. Similarly, for angle sensing, the sensitivity is expressed as $S_{\delta }=\frac{\Delta \delta }{\Delta \theta }$, in which Δδ corresponds to the variation in spin shift induced by an incident angle change Δθ.

Sulfur dioxide (SO_2_), a major atmospheric pollutant, poses significant threats to human health, animal health, and ecosystem stability. The minimum lethal concentration of human exposure to SO_2_ is 400 ppm for 1 min [39]. Therefore, it is of crucial importance to monitor the concentration of SO_2_ in the air. The refractive index of a mixture of SO_2_ and air, $n_{mix}$, can be expressed as a linear combination of the refractive indices of air ($n_{air}$) [40] and SO_2_ ($n_{{SO}_{2}}$) [41] weighted by the SO_2_ concentration ($C_{{SO}_{2}}$) [42]:

(15) $$n_{mix}=n_{air}\left(1-C_{{SO}_{2}}\right)+n_{{SO}_{2}}C_{{SO}_{2}}$$

In this work, we focus on detecting SO_2_ concentrations ranging from 200 to 400 ppm, corresponding to a refractive index range of 1.00034637–1.00041453 for the gas mixture.

**Figure 5:**
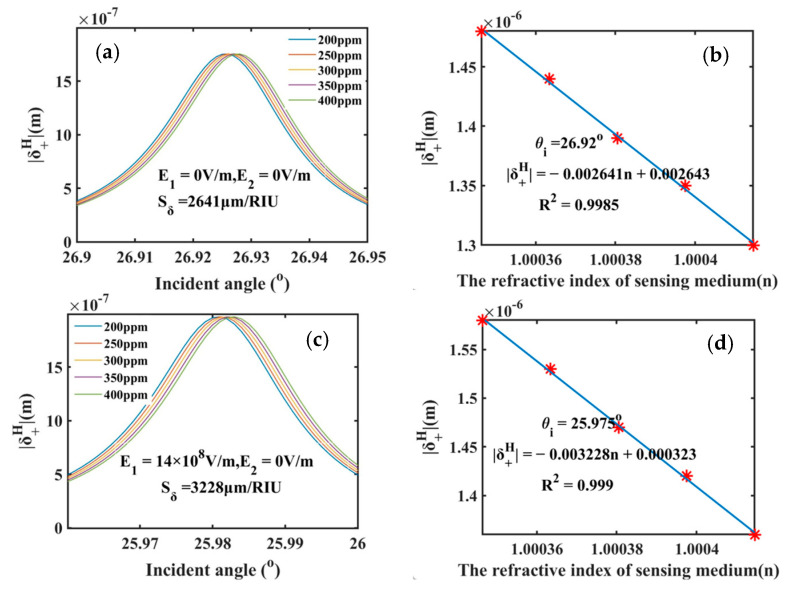
The refractive index sensor based on the SPR structure for detecting the SO_2_ concentrations and the sensing performance. Here, (**a**,**b**) corresponds to the condition without an electric field; (**c**,**d**) is under the field condition of $E_{1}$-only.

Figure 5a shows the spin shifts of SPR sensor (without electric field) versus the incident angle for various SO_2_ concentrations. As the SO_2_ concentration increases from 200 to 400 ppm, the curve exhibits observable movement towards higher incident angle, and the maximal value of spin shift remains constant. This behavior enables the detection of SO_2_ concentrations through the spin shift measurements at fixed incident angles. At a fixed incident angle of 26.92°, the spin shift as a function of the gas mixture refractive index in the range from 1.00034637 to 1.00041453 is plotted in Figure 5b, which basically presents a linear relation. From the slope of the linear-fit curve in Figure 5b, the intensity sensitivity of 2641 μm/RIU is obtained for the zero-field mode. In the same way, when applying only $E_{1}$, the sensitivity is enhanced to 3228 μm/RIU, approximately 4–5 times higher than that of VO_2_-based thermal-controlled PSHE multi-functional sensor [26]. This significant enhancement of sensitivity highlights the superior performance of our designed electric-field-controlled PSHE sensor for gas pollutant detection.

For the SPR structure with the Kretschmann configuration, angular interrogation is a common method for tracking refractive index variations [43]. However, when detecting SO_2_ concentrations of 200–400 ppm (corresponding to a refractive index change of just 0.00006816 RIU), the resulting shift of SPR angle is merely 0.002°, yielding a sensitivity of only 29.3°/RIU. In contrast, PSHE-based sensors can overcome this low-sensitivity limitation by translating small refractive index changes into measurable spin shifts. Our results demonstrate that the same refractive index variation produces clearly detectable spin shift signals, indicating the superior sensitivity of PSHE detection compared to conventional angular interrogation. This enhanced sensing performance for ultra-small refractive index changes represents a significant advancement for detecting trace gas pollutants.

**Figure 6:**
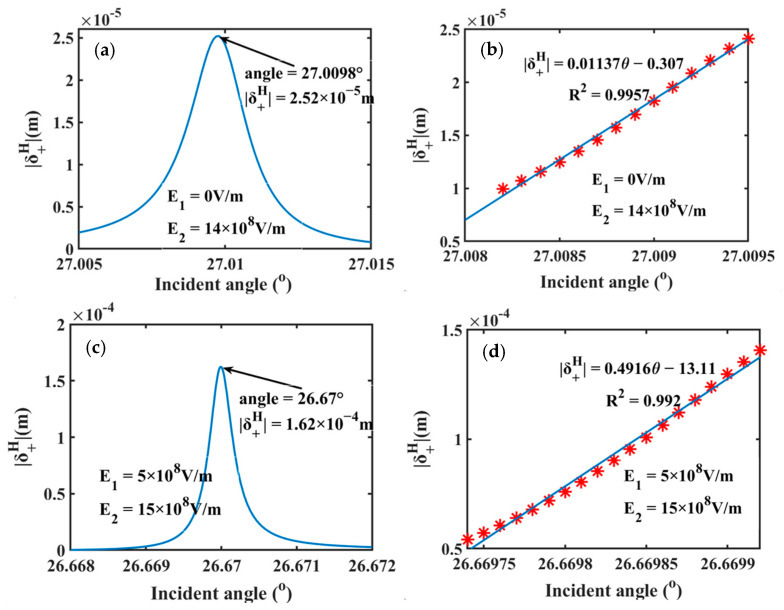
The angle sensor based on the SPR structure and the sensing performance. Here, (**a**,**b**) corresponds to the field condition of $E_{2}$-only; (**c**,**d**) is under the condition of dual-field control.

Angle sensors, as critical components for high-precision measurement, play a vital role in modern industrial and manufacturing systems [44,45]. Driven by the advancements in precision manufacturing and intelligent equipment, these sensors are undergoing significant evolution towards enhanced sensitivity and miniaturized design. As depicted in Figure 6, under the conditions of $E_{1}$-only applied and dual-field control, the spin shifts in the SPR structure are highly sensitive to the small angle variations. This extraordinary sensitivity enables the angle resolution at sub-millimeter level (<0.001°), creating new opportunities for the development of precision measurement technologies. Figure 6a shows the dependence of spin shifts on the incident angle (with $E_{1}$-only applied), reaching a peak value of 252 μm at 27.0098°. Within the angle range of 27.0082°–27.0095°, the spin shifts of PSHE present a linear relationship with incident angle ($R^{2}$ = 0.9957). This linear relation allows direct conversion of angle variations into the measurable PSHE spin shifts, and the angle sensitivity of 0.01137 m/° is achieved. As displayed in Figure 6b, the dual-field control can further enhance this angle sensitivity to 0.4916 m/° ($R^{2}$ = 0.992) within the linear angle range of 26.66975°–26.66990°. This represents a 43-fold improvement over the $E_{1}$-only configuration and surpasses the sensing performance of conventional angle sensors based on asymmetry coupling of the square- and L-shaped structure (0.1 m/°) by a factor of 4.9 [46]. These results are obtained by setting the refractive index of sensing medium layer to be 1. It should be noted that varying the sensing medium’s refractive index enables dynamic adjustment of angle detection ranges.

The angle detection range and corresponding sensitivity for the $E_{2}$-only and dual-field control conditions are summarized in Table 1 and Table 2, respectively, with variable refractive index of sensing medium (*n*). As *n* increases from 1 to 1.02, the angle detection range moves toward higher angle. For instance, under the condition of $E_{2}$-only application, the angle detection range shifts from 27.0082°–27.0096° to 27.6350°–27.6363°, corresponding to a Δθ of 0.63°. However, when *n =* 1.02, the sensitivity drops sharply to 0.30 × 10^4^ μm/°. Due to this significant decline, further calculations for higher *n* are not pursued. Under the dual-field control, a similar shift in the angle detection range (Δθ ~ 0.62°) occurs, but unlike the $E_{2}$-only applied case, the higher sensitivity is preserved. The above analysis demonstrates that the angle detection range can be dynamically tuned in practical applications through the optimal selection of sensing medium’s refractive index.

## 4. Conclusions

In summary, we have theoretically demonstrated an electric-field-controlled dynamic modulation scheme for the PSHE in a SPR configuration. The proposed structure comprises a LN prism, Ag thin film, and sensing medium, with independent electric fields ($E_{1}$ and $E_{2}$) applied to the LN prism and Ag film, respectively. The Pockels EO effect facilitates the flexible tuning of refractive indices in both LN prism and Ag film, thereby achieving the dynamic modulation of PSHE in this SPR system. Notably, under the dual-field control, the maximal value of PSHE spin shifts is enhanced by two orders of magnitude when compared to the case without electric field. Furthermore, based on the various operational modes of external electric fields, the SPR structure can realize the multi-functional sensing (refractive index and angle detections) with superior performance. These findings open new opportunities for active PSHE manipulation and the development of multi-functional optical sensing.

## Figures and Tables

**Table 1:** The angle detection range and sensitivity under the condition of $E_{2}$-only application.

Refractive Index of Sensing Medium (*n*)	Angle Detection Range (*θ*)	$\mathrm{Angle}\;\mathrm{Sensitivity}\;(S_{\theta }$)
1.0000	27.0082°–27.0095°	1.13 × 10^4^ μm/°
1.0001	27.0113°–27.0126°	1.13 × 10^4^ μm/°
1.0005	27.0239°–27.0252°	1.13 × 10^4^ μm/°
1.001	27.0395°–27.0408°	1.13 × 10^4^ μm/°
1.005	27.1647°–27.2660°	1.00 × 10^4^ μm/°
1.01	27.3216°–27.3229°	0.89 × 10^4^ μm/°
1.02	27.6350°–27.6363°	0.30 × 10^4^ μm/°

**Table 2:** The angle detection range and sensitivity under the condition of dual-field control.

Refractive Index of Sensing Medium (*n*)	Angle Detection Range (*θ*)	$\mathrm{Angle}\;\mathrm{Sensitivity}\;(S_{\theta }$)
1.00000	26.66974°–26.66992°	4.91 × 10^5^ μm/°
1.00001	26.67005°–26.67023°	4.95 × 10^5^ μm/°
1.00005	26.67129°–26.67147°	5.07 × 10^5^ μm/°
1.0001	26.67284°–26.67302°	5.20 × 10^5^ μm/°
1.0005	26.68520°–26.68538°	5.64 × 10^5^ μm/°
1.001	26.70055°–26.70083°	5.31 × 10^5^ μm/°
1.002	26.73144°–26.73162°	6.49 × 10^5^ μm/°
1.01	26.97895°–26.97900°	9.28 × 10^6^ μm/°
1.02	27.28925°–27.28943°	4.87 × 10^5^ μm/°

## Data Availability

The data presented in this study are available on request from the corresponding author.
